# Risk factors of uncontrolled periprosthetic knee joint infection after two-stage reimplantation

**DOI:** 10.1186/s43019-020-00041-8

**Published:** 2020-05-19

**Authors:** Du-Han Kim, Ki-Cheor Bae, Dong-Wan Kim, Byung-Chan Choi

**Affiliations:** Department of Orthopedic Surgery, Keimyung University School of Medicine, 1035, Dalgubeol-daero, Dalseo-gu, Daegu, 42601 South Korea

**Keywords:** Knee, Arthroplasty, Infection, Complications, Reimplantation

## Abstract

**Background:**

Periprosthetic infection after total knee arthroplasty is a challenging problem, and physicians should identify risk factors to decrease recurrence. However, risk factors for reinfection with two-stage reimplantation have not yet been well established. The purpose of this study was to assess treatment outcomes of subsequent two-stage knee reimplantation and identify risk factors for uncontrolled periprosthetic knee joint infections.

**Methods:**

We retrospectively reviewed 70 knees diagnosed with a periprosthetic knee joint infection treated with two-stage reimplantation between September 2011 and October 2016 at our institution. Patients in the controlled infection group (group C) required no further medication or surgical treatment within 2 years after reimplantation. Patients in the uncontrolled infection group (group U) displayed symptoms of active infection after resection arthroplasty or were reinfected after two-stage reimplantation. We compared group C and group U, and analyzed potential risk factors for uncontrolled prosthetic joint infection (PJI).

**Results:**

Of 70 knees included in this analysis, 53 (75.7%) were clinically deemed free from infection at the latest follow-up. The remaining 17 knees (24.3%) required additional surgical procedures after two-stage reimplantation. Demographics were not statistically significantly different between the two groups. Wound complications were statistically more frequent in group U (*p* = 0.030). Pre-reimplantation C-reactive protein (CRP) was statistically different between groups C and U (0.44 and 1.70, respectively, *p* = 0.025). Among the cultured microorganisms, fungus species were statistically more frequently detected in group U compared with group C (*p* = 0.031).

**Conclusions:**

The reinfection rate of our two-stage reimplantation protocol was 24.3% in the included cases. Wound complications, higher pre-reimplantation CRP levels, and fungus species were statistically more common in group U compared with group C. Our findings will help in counseling patients and physicians to understand that additional caution may be required when treating PJI if the aforementioned risk factors are present.

**Level of evidence:**

IV, case series.

## Background

Remarkable outcomes have been achieved in many patients who underwent total knee arthroplasty (TKA), including improved quality of life [1]. Importantly, however, some patients treated with primary TKA do not achieve optimal outcomes, and total failure requiring revision arthroplasty may occur. Prosthetic joint infection (PJI) is one of the most common causes of TKA failure, occurring in approximately 2% of patients [2]. As the mean age of those who undergo TKA decreases and people live longer, the number of people suffering from PJI after TKA is rapidly increasing.

For cases of acute infections with a stable implant and adequate soft tissue mass, treatments designed to retain the existing implant are recommended; however, treatments are more complicated in chronic infections [3]. For patients with a chronic, peri-knee implant infection, two-stage reimplantation is preferred as this approach is associated with the highest chance to both eradicate the infection and provide patients with a functional and pain-free TKA [4]. However, numerous two-stage reimplantation protocols have been reported and treatment results vary by surgeon [3, 5].

Risk factors associated with the occurrence of infection after primary TKA have been extensively studied and reported in the literature [6–10]. Risk factors include patient-associated factors (e.g., young age, male sex, high body mass index (BMI), diabetes mellitus (DM), preoperative comorbidities, previous knee surgeries, rheumatoid arthritis) and surgical factors (e.g., operative time, surgeon experience and patient volume, hospital experience and patient volume) [11, 12]. However, risk factors for reinfection with two-stage reimplantation have not yet been well established. Therefore, the purpose of this study was to assess outcomes of two-stage reimplantation for PJI and to identify risk factors associated with failure after two-stage reimplantation knee arthroplasty.

## Methods

**Fig. 1:**
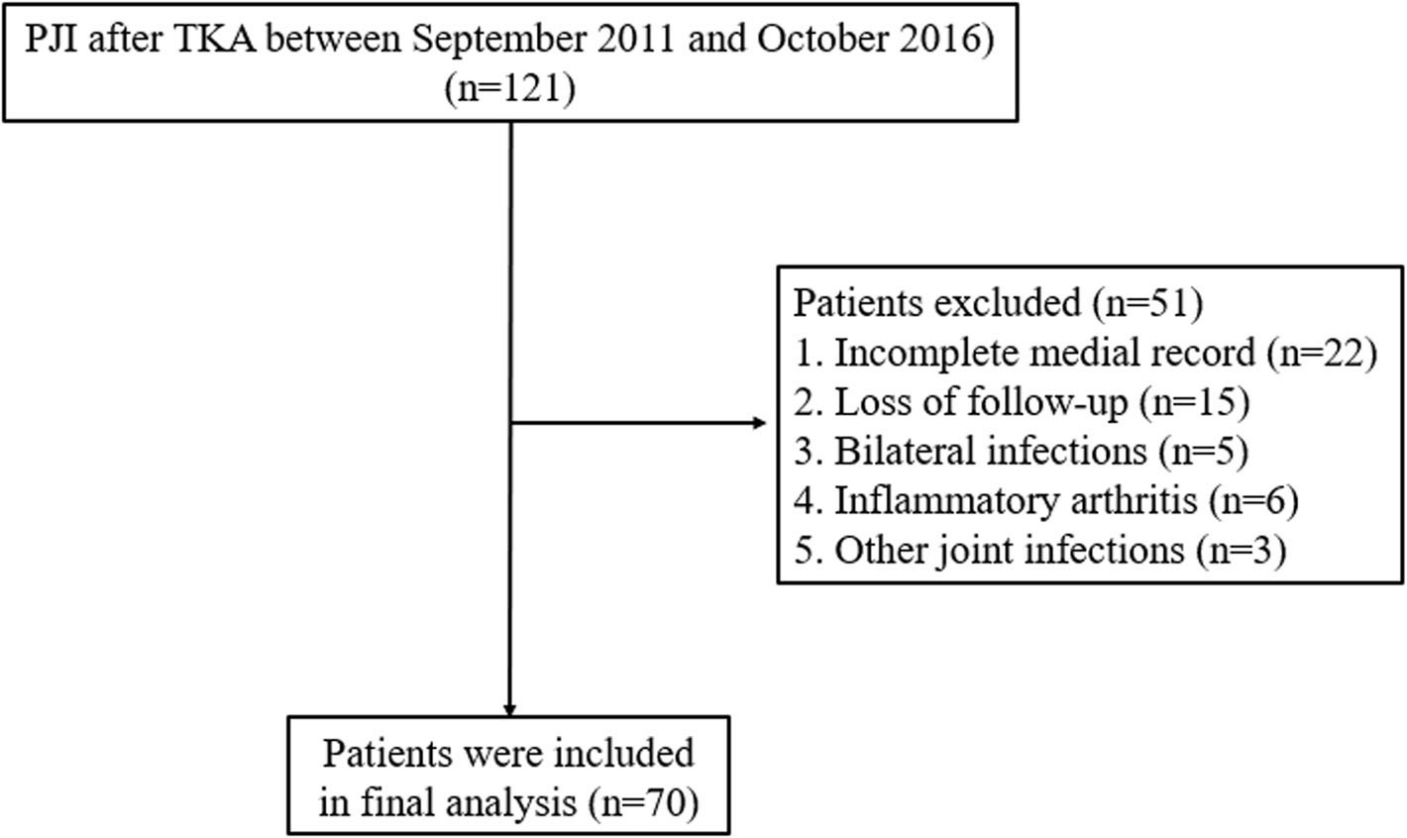
Patient flow chart. PJI prosthetic joint infection, TKA total knee arthroplasty

We retrospectively reviewed 121 knees diagnosed with a PJI after TKA between September 2011 and October 2016 at our institution. This study was approved by our institutional review board (IRB No. 2019-05-001). PJI was treated with two-stage reimplantation by a single surgeon and a uniform protocol. Inclusion criteria follow the Musculoskeletal Infection Society (MSIS) guidelines for PJI [13]. Patients treated with alternative approaches or those who refused reimplantation for personal issues were excluded. Patients with incomplete medical records, less than 2 years of follow-up, or bilateral cases were also excluded (Fig. 1).

A total of 70 knees met the inclusion criteria and were retrospectively reviewed. Patients were placed in the controlled infection group (group C) if they required no further medication or surgical treatment within 2 years after reimplantation. Patients were placed in the uncontrolled infection group (group U) if they presented with symptoms of active infection after resection arthroplasty or were reinfected after two-stage reimplantation.

### Data collection

Multiple potential predictive variables were collected from the medical record, including demographic data (e.g., age, gender, BMI, affective side, symptom duration), comorbidities (e.g., hypertension, DM, liver cirrhosis, chronic kidney disease, cancer history, previous infection history, anticoagulant abuse), and operation-related factors (wound complications, hemarthrosis, transfusion, deep vein thrombosis). Clinical outcomes were assessed using the Korean Knee Score (KKS). C-reactive protein (CRP) and the erythrocyte sedimentation rate (ESR) were retrospectively evaluated pre-resection (first stage) and pre-reimplantation (second stage). Cultured microorganisms were classified into one of five groups and analyzed: methicillin-sensitive organisms; methicillin-resistant organisms; fungus species; *Pseudomonas* species; and other species.

### First stage procedure: resection arthroplasty

**Fig. 2:**
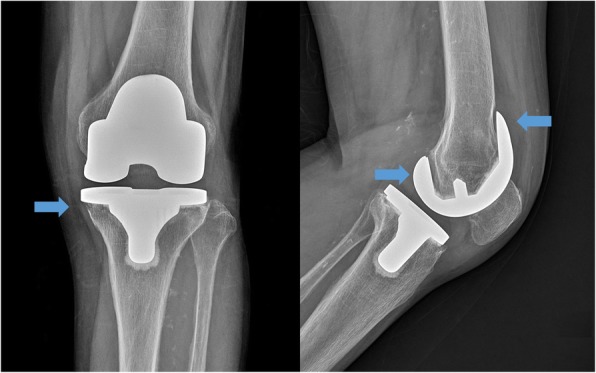
Plain radiograph from a 76-year-old female 3 years after primary total knee arthroplasty. Blue arrows indicate bone resorption around the femoral prosthesis and medial condyle of tibia

**Fig. 3:**
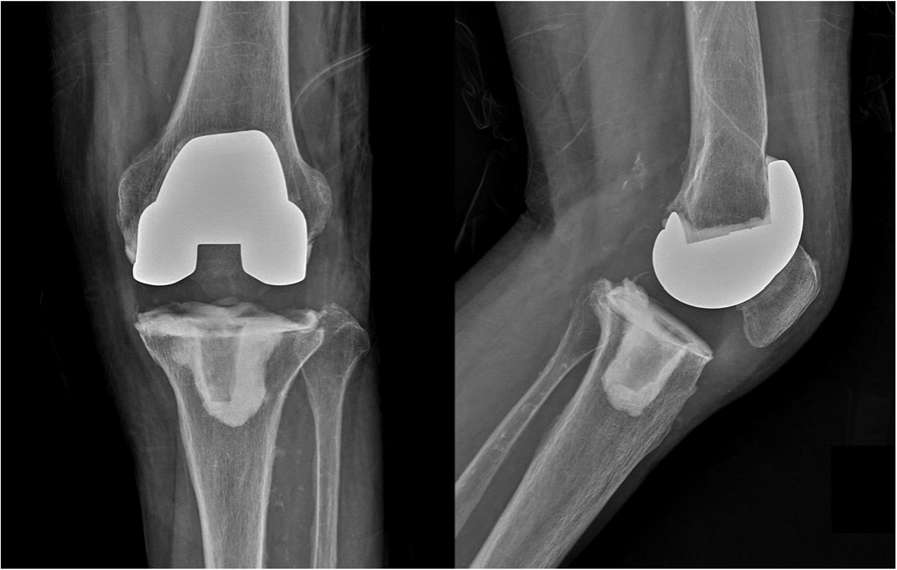
Postoperative plain radiograph of resection arthroplasty. The infected prosthesis was removed, and antibiotic-mixed cement was placed on the articular side of the femur and tibia

The first stage involved resection arthroplasty with antibiotic-loaded cement spacer (Fig. 2). All patients with PJI underwent resection arthroplasty with the removal of all prosthetic components and cement as well as debridement of necrotic tissue. Bone cement and vancomycin were mixed at a mass ratio of 10:1. Antibiotic-mixed cement was placed on each articular side for two reasons: to cover the bone defect and prevent soft tissue contracture; and to provide direct local delivery of high doses of antibiotics, avoiding systemic toxicity [14]. We reuse the femoral implant after the autoclaving process, and the femoral implant with gentamicin-mixed cement (CMW; DePuy Synthes, Warsaw, IN, USA) is placed on the articular side of the femur. A high cross-linked polyethylene liner (TC3 knee system; DePuy Synthes) was placed between the femoral component and bone cement on the tibial side, which acted like a bearing. A drain was left in the knee joint and aided in evaluating the knee joint status (Fig. 3).

### Management between first stage and second stage

All patients underwent 6 weeks of parenteral drugs, selected based on sensitivity to intraoperative-cultured pathogens. At postoperative week 6, if the patient still showed no sign of infection, parenteral drugs were changed to oral medicines for 4 weeks and patients were closely monitored in outpatient clinics. The timing of the second-stage reimplantation was based on clinical condition and laboratory data. Reimplantation was performed after 2 weeks of the antibiotic holiday without elevation of the ESR and CRP. In patients who did not confirm normal laboratory data, we performed reimplantation according to the clinical condition combined with a trend of decreased ESR and CRP levels after discontinuing oral medicines.

### Second stage procedure: reimplantation

**Fig. 4:**
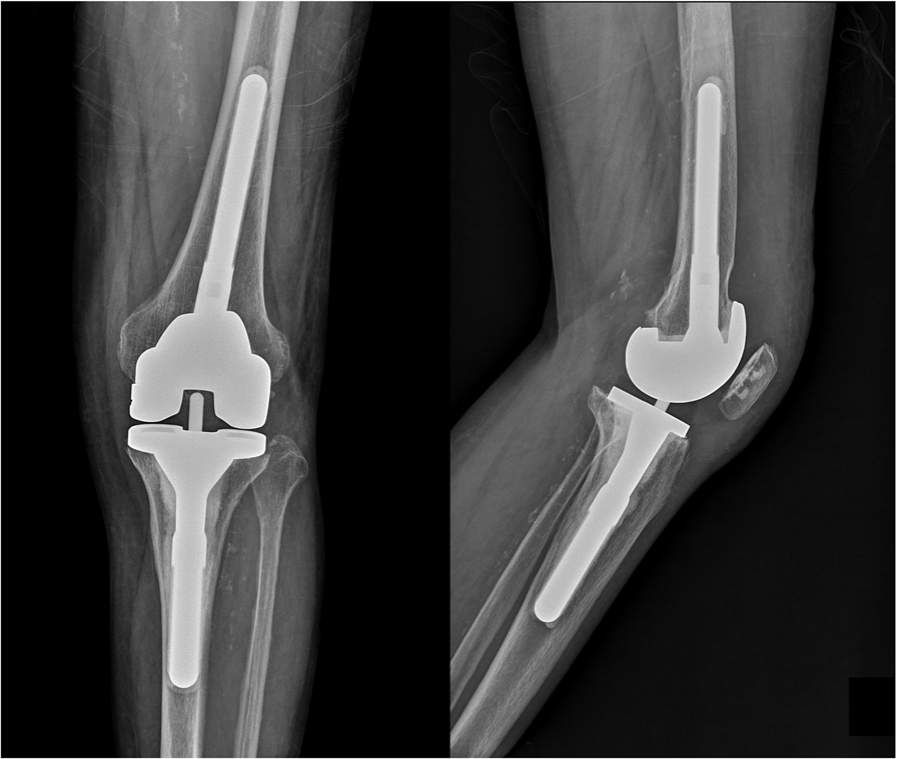
Post-reimplantation plain radiograph

At postoperative week 12, reimplantation was performed. Meticulous debridement was conducted again. If intraoperative findings revealed suspicion of infection (e.g., purulent exudates), the protocol dictated a return to the first stage with the replacement of the cement spacer. If intraoperative findings suggested eradication of infection, revision arthroplasty was planned. Any debris or soft tissue that showed loss of viability was debrided. The femoral prosthesis, polyethylene liner, and cement were removed and the tibial and femoral medulla were curetted. All patients were treated with a standard rotating hinge prosthesis (TC3; DePuy Synthes). Metal augmentation and cement were used to cover bone defects and a stem was used to provide stability to the prosthesis (Fig. 4). As the previous infection was considered controlled, first-generation cephalosporins were administered for 6 days postoperatively. Patients were followed in the outpatient clinic every 3 months.

### Statistical analysis

Analyses were performed using SPSS version 21.0 (SPSS Incorporated, Chicago, IL, USA). Categorical and continuous variables were expressed as the count and the mean ± standard deviation (range), respectively. Proportional hazard regression univariate analysis was performed to assess the association of clinical covariates with the risk of uncontrolled infection. The Mann–Whitney test or chi-square/Fisher’s exact test was used to compare patient characteristics, comorbidities, type of cultured pathogens, and laboratory results between groups C and U. *p* < 0.05 was considered statistically significant.

## Results

**Fig. 5:**
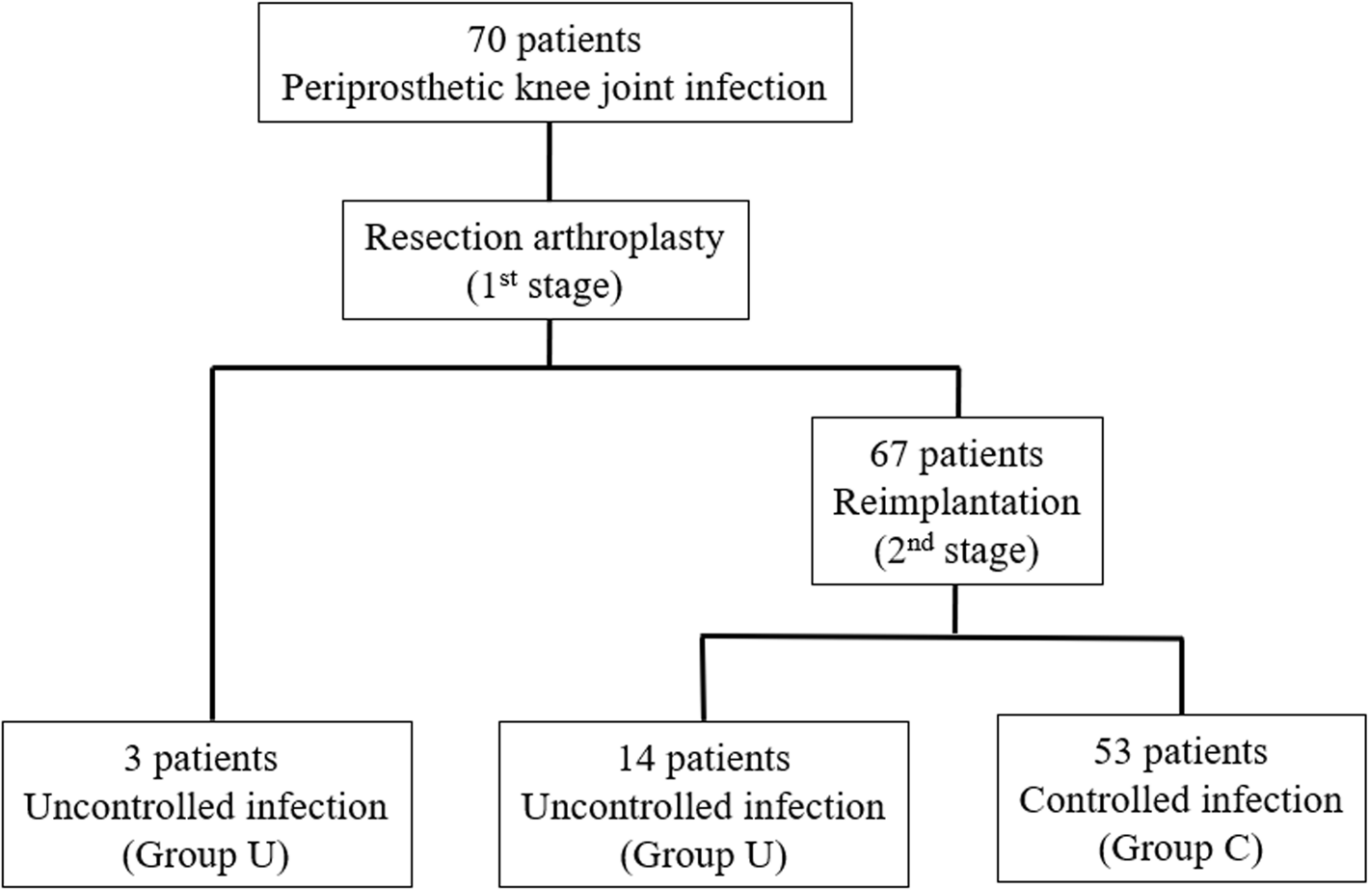
Patient flow chart for the controlled infection group (group C) and the uncontrolled infection group (group U)

Of the 70 knees included in this analysis, 67 were deemed clinically stable after resection arthroplasty; the remaining three required additional surgical treatment due to remaining infection and were classified as the first-stage failure group. Of the 67 clinically stable knees, 53 were clinically deemed free from infection after reimplantation and assigned to group C. The remaining 14 knees were reinfected after two-stage reimplantation; each retreated with two-stage reimplantation (Fig. 5).

There were no statistically significant differences in demographics (i.e., mean age, male-to-female sex ratio, height, weight, direction of affected site, duration of follow-up) between groups C and U. Clinical outcome at final follow-up assessed using the KKS was 68.0 in group C and 59.5 in group U, a difference that was not statistically significant (*p* = 0.180) (Table 1).

**Table 1:** Demographic data for patients in group C and group U

Variable	Group C (*n* = 53)	Group U (*n* = 17)	*p* value
Age	69.72 ± 6.46	66.88 ± 6.67	0.121
Sex			1.000
Male	9 (17.0%)	3 (17.6%)	
Female	44 (83.0%)	14 (82.4%)	
Body mass index	25.5 ± 8.4	24.4 ± 9.2	0.438
Affected side			0.525
Right	27 (50.9%)	7 (41.2%)	
Left	26 (49.1%)	10 (58.8%)	
Symptom duration (months)	7.3 ± 11.6	7.8 ± 11.0	0.715
Interval between TKA and resection arthroplasty (months)	24.2 ± 21.4	16.9 ± 11.8	0.854
Duration of follow-up (months)	32.6 ± 8.0	33.7 ± 7.7	0.629
Previous history of knee surgery	9 (17.0%)	1 (5.9%)	1.000
Final Korean Knee Score	68.0 ± 12.1	59.5 ± 10.3	0.180

Data presented as mean ± standard deviation or *n* (%). *Group C* controlled infection group, *Group U* uncontrolled infection group, *TKA* total knee arthroplasty

Univariate analysis for risk factors revealed that wound complications (e.g., dehiscence, discharge) occurred significantly more frequently in group U (*n* = 8) compared with group C (*n* = 11) (*p* = 0.03). However, the prevalence of other comorbidities was not significantly different between group C and group U (Table 2).

**Table 2:** Univariate analysis of selected variables for patients in group C and group U

Variable	Group C (*n* = 53)	Group U (*n* = 17)	*p* value
Wound complications	11 (20.8%)	8 (47.1%)	0.030*
Hemarthrosis	0 (0%)	2 (11.8%)	0.055
Transfusion	32 (60.4%)	10 (58.8%)	0.975
Deep vein thrombosis	7 (13.2%)	5 (29.4%)	0.069
Hypertension	30 (56.6%)	10 (58.8%)	0.813
Diabetes mellitus	19 (35.8%)	6 (35.3%)	0.629
Liver cirrhosis	1 (1.9%)	0 (0%)	1.000
Chronic kidney disease	3 (5.7%)	0 (0%)	1.000
Cancer history	3 (5.7%)	1 (5.9%)	0.270
Previous infection of other organs	9 (17.0%)	1 (5.9%)	0.433
Anticoagulant	14 (26.4%)	6 (35%)	0.069

Data presented as *n* (%). *Group C* controlled infection group, *Group U* uncontrolled infection group

*p < 0.05 considered statistically significant

Pre-reimplantation CRP was significantly higher in group U than group C (1.70 ± 2.85 and 0.44 ± 0.47, respectively; *p* = 0.025). Pre-resection CRP, pre-resection ESR, and pre-implantation ESR were not significantly different between group C and group U (*p* = 0.205, *p* = 0.593, and *p* = 0.509, respectively). The presence of fungus species using culture tests was statistically more frequent in group U compared with group C (*p* = 0.031). The presence of methicillin-resistant or methicillin-sensitive organisms, *Pseudomonas*, and other species was not significantly different between groups (*p* = 0.882, *p* = 0.517, *p* = 0.327, and *p* = 0.572, respectively) (Table 3).

**Table 3:** Univariate analysis of laboratory results and identification of pathogens between group C and group U

Variables	Group C (*n* = 53)	Group U (*n* = 17)	*p* value
Pre-resection CRP	5.87 ± 6.74	3.11 ± 3.33	0.205
Pre-resection ESR	80.48 ± 32.88	75.53 ± 29.36	0.593
Pre-reimplantation CRP	0.44 ± 0.47	1.70 ± 2.85	0.025*
Pre-reimplantation ESR	42.93 ± 22.59	50.71 ± 33.72	0.509
Identification of pathogen	34 (64.2%)	12 (70.6%)	0.111
Methicillin-resistant organisms	19 (35.8%)	6 (35.3%)	0.882
Methicillin-sensitive organisms	11 (20.8%)	2 (11.8%)	0.517
Fungal species	2 (3.8%)	3 (17.6%)	0.031*
Pseudomonal species	1 (1.9%)	1 (5.9%)	0.327
Other organisms	1 (1.9%)	0 (0%)	0.572

Data presented as mean ± standard deviation or *n* (%). *CRP* C-reactive protein, *ESR* erythrocyte sedimentation rate, *Group C* controlled infection group, *Group U* uncontrolled infection group

*p < 0.05 considered statistically significant

## Discussion

In this study, we found that 17 out of 70 patients (24.3%) developed recurrent infections after our two-stage reimplantation. These outcomes are similar to findings of other studies in the literature (i.e., incidence of infection recurrence ranges from 10% to 28%) [4, 15, 16]. Additionally, the univariate analysis identified pre-reimplant CRP, wound complications, and fungal species as risk factors for uncontrolled PJI following two-stage reimplantation.

Several risk factors for PJI have been published; however, little is known about potential risk factors for PJI following failed two-stage reimplantation arthroplasty. Sakellariou et al. identified patients with a background of inflammatory arthritis or those who were immunocompromised to be at an increased risk for reinfection [16]. Also, in an analysis of 368 patients by Kubista et al., it was noted that the strongest positive predictors of treatment failure included chronic lymphoedema, and revision between resection and definitive reimplantation and patients treated with intravenously administered cefazolin had a significant reduction in recurrent infection rates [4]. Watts et al. reported that morbidly obese patients (i.e., BMI ≥ 40 kg/m^2^) had significantly higher rates of subsequent revision (hazard ratio, 4.45), reinfection (hazard ratio, 4.86), and reoperation (hazard ratio, 4.37) following two-stage TKA revision for PJI when compared with a matched cohort of nonobese patients (i.e., BMI < 30 kg/m^2^) [17]. Similar studies were also conducted in joints other than the knee. Jhan et al. evaluated 62 patients with chronic PJI of the hip joint treated with two-stage reimplantation and found that obesity, liver cirrhosis, Gram-negative organisms, and presence of a sinus tract were significantly related to the risks of failure [5]. In our study, the prevalence of DM and obesity, and the use of anticoagulant (also a known risk factor of PJI), were higher in group U compared with group C; however, this difference was not statistically significant.

Although interpreting laboratory tests is crucial, there is ongoing debate and controversy relating to the role of laboratory results in PJI. Ghanem et al. studied 109 patients with infected TKAs who underwent two-stage revision and were unable to define an absolute CRP or ESR threshold for infection eradication despite a 21% (23 of 109) reinfection rate at an average of 2 years [18]. Stambough et al. analyzed 291 cases of PJI and suggested that the percent change in serum ESR and CRP inflammatory markers before and after two-stage reimplantation for PJI was not associated with the reinfection risk when controlling for ASA class [19]. Furthermore, this group concluded that inflammatory markers provide no additional diagnostic accuracy for determining the timing of reimplantation. Lee and Chen reported in a systematic review and meta-analysis that no single marker was superior to all of the others. Because none is likely sufficient to confirm control of infection after the first stage of the two-stage protocol for PJI, they suggested that multiple tools are needed for ensuring infection eradication. [20]. In this present study, there was no significant difference in pre-resection ESR results between both groups; however, the pre-resection CRP level was lower in group U than in group C. Similar to our study, Petrikkos et al. noted that both serum procalcitonin and CRP levels were lower in patients with fungal infections than in those with bacterial infection [21].

Several studies of uncontrolled PJI and microorganisms have been published. Earlier studies reported higher failure rates in periprosthetic infection when methicillin-resistant bacteria are present [22–24]. Importantly, we note that these studies included patients with hip PJI treated with several different strategies. Kubista et al. were unable to detect a statistically significant difference in recurrence rates between patients with confirmed infection with methicillin-sensitive and methicillin-resistant organisms [4]. The presence of *Enterococcus* or *Streptococcus* species was also associated with a higher risk of failure in a study by Citak et al. [25] Furthermore, several authors reported that polymicrobial periprosthetic infections were at an increased risk for recurrence infection [16, 26, 27]. Here, we report no significant differences between methicillin-sensitive and methicillin-resistant species, an observation consistent with studies by Kubista et al. [4] This observation is thought to be the result of vancomycin usage, which spreads from cement to kill methicillin-resistant or methicillin-sensitive species. Recently, attempts have been made to mix amphotericin B or voriconazole into cement, drugs which are effective against fungal species leading to favorable outcomes [28]. Further studies on the heat stability and local spread of antimicrobial agents are needed.

The strengths of this study include that it was performed in a homogeneous group of patients who underwent surgery by a single surgeon. Also, this was the first report of risk factors for uncontrolled infection after two-stage reimplantation in Korean patients. However, our study has several limitations: a retrospective design and relatively small cohort; our institution is a tertiary referral medical center, so primary arthroplasty was performed by various surgeons and methods—prostheses were also from different companies and biology of the knee can be ruined in various ways, which may affect the final treatment result; intraoperative frozen section histopathology, which yields a high diagnostic accuracy matched with MSIS criteria, was not routinely checked; and we did not check the comorbidity scores. Further well-designed prospective clinical studies are needed to confirm our results.

## Conclusion

The reinfection rate of our two-stage reimplantation protocol showed 24.3% in the included cases. Wound complications, higher pre-reimplantation CRP levels, and fungus species were statistically more common in group U compared with group C. Our findings will help in counseling patients and physicians to understand that additional caution may be required when treating PJI if the aforementioned risk factors are present.

## Data Availability

The datasets used and/or analyzed during the current study are available from the corresponding author on reasonable request.

## Abbreviations

CRP: C-reactive protein
ESR: Erythrocyte sedimentation rate
KKS: Korean Knee Score
MSIS: Musculoskeletal Infection Society
PJI: Prosthetic joint infection
TKA: Total knee arthroplasty

## References

[1] Ranawat C (2002). History of total knee replacement. J South Orthop Assoc.

[2] Abblitt WP, Chan EW, Shinar AA (2018). Risk of periprosthetic joint infection in patients with multiple arthroplasties. J Arthroplast.

[3] Kuiper JW, Willink RT, Moojen DJ, van den Bekerom MP, Colen S (2014). Treatment of acute periprosthetic infections with prosthesis retention: review of current concepts. World J Orthop.

[4] Kubista B, Hartzler RU, Wood CM, Osmon DR, Hanssen AD, Lewallen DG (2012). Re-infection after two-stage revision for periprosthetic infection of total knee arthroplasty. Int Orthop.

[5] Jhan SW, Lu YD, Lee MS, Lee CH, Wang JW, Kuo FC (2017). The risk factors of failed reimplantation arthroplasty for periprosthetic hip infection. BMC Musculoskelet Disord.

[6] Mortazavi SM, Vegari D, Ho A, Zmistowski B, Parvizi J (2011). Two-stage exchange arthroplasty for infected total knee arthroplasty: predictors of failure. Clin Orthop Relat Res.

[7] Mortazavi SM, Schwartzenberger J, Austin MS, Purtill JJ, Parvizi J (2010). Revision total knee arthroplasty infection: incidence and predictors. Clin Orthop Relat Res.

[8] Minnema B, Vearncombe M, Augustin A, Gollish J, Simor AE (2004). Risk factors for surgical-site infection following primary total knee arthroplasty. Infect Control Hosp Epidemiol.

[9] Bongartz T, Halligan CS, Osmon DR, Reinalda MS, Bamlet WR, Crowson CS (2008). Incidence and risk factors of prosthetic joint infection after total hip or knee replacement in patients with rheumatoid arthritis. Arthritis Rheum.

[10] Parvizi J, Ghanem E, Sharkey P, Aggarwal A, Burnett RS, Barrack RL (2008). Diagnosis of infected total knee: findings of a multicenter database. Clin Orthop Relat Res.

[11] Peersman G., Laskin R., Davis J., Peterson Margaret (2001). Infection in Total Knee Replacement. Clinical Orthopaedics and Related Research.

[12] Kim TW, Makani A, Choudhury R, Kamath AF, Lee GC (2012). Patient-reported activity levels after successful treatment of infected total knee arthroplasty. J Arthroplast.

[13] Parvizi J, Zmistowski B, Berbari EF, Bauer TW, Springer BD, Della Valle CJ (2011). New definition for periprosthetic joint infection: from the Workgroup of the Musculoskeletal Infection Society. Clin Orthop Relat Res.

[14] Jia YT, Zhang Y, Ding C, Zhang N, Zhang DL, Sun ZH (2012). Antibiotic-loaded articulating cement spacers in two-stage revision for infected total knee arthroplasty: individual antibiotic treatment and early results of 21 cases. Chin J Traumatol.

[15] Bejon P, Berendt A, Atkins BL, Green N, Parry H, Masters S (2010). Two-stage revision for prosthetic joint infection: predictors of outcome and the role of reimplantation microbiology. J Antimicrob Chemother.

[16] Sakellariou VI, Poultsides LA, Vasilakakos T, Sculco P, Ma Y, Sculco TP (2015). Risk factors for recurrence of periprosthetic knee infection. J Arthroplast.

[17] Watts CD, Wagner ER, Houdek MT, Osmon DR, Hanssen AD, Lewallen DG (2014). Morbid obesity: a significant risk factor for failure of two-stage revision total knee arthroplasty for infection. J Bone Joint Surg Am.

[18] Ghanem E, Azzam K, Seeley M, Joshi A, Parvizi J (2009). Staged revision for knee arthroplasty infection: what is the role of serologic tests before reimplantation?. Clin Orthop Relat Res.

[19] Stambough JB, Curtin BM, Odum SM, Cross MB, Martin JR, Fehring TK (2019). Does change in ESR and CRP guide the timing of two-stage arthroplasty reimplantation?. Clin Orthop Relat Res.

[20] Lee YS, Chen AF (2018). Two-stage reimplantation in infected total knee arthroplasty. Knee Surg Relat Res.

[21] Petrikkos GL, Christofolopoulou SA, Tentolouris NK, Charvalos EA, Kosmidis CJ, Daikos GL (2005). Value of measuring serum procalcitonin, C-reactive protein, and mannan antigens to distingush fungal from bacterial infections. Eur J Clin Microbiol Infect Dis.

[22] Salgado CD, Dash S, Cantey JR, Marculescu CE (2007). Higher risk of failure of methicillin-resistant *Staphylococcus aureus* prosthetic joint infections. Clin Orthop Relat Res.

[23] Parvizi J, Azzam K, Ghanem E, Austin MS, Rothman RH (2009). Periprosthetic infection due to resistant staphylococci: serious problems on the horizon. Clin Orthop Relat Res.

[24] Mortazavi SM, Molligan J, Austin MS, Purtill JJ, Hozack WJ, Parvizi J (2011). Failure following revision total knee arthroplasty: infection is the major cause. Int Orthop.

[25] Citak M, Friedenstab J, Abdelaziz H, Suero EM, Zahar A, Salber J (2019). Risk factors for failure after 1-stage exchange total knee arthroplasty in the management of periprosthetic joint infection. J Bone Joint Surg Am.

[26] Jackson Walter O., Schmalzried Thomas P. (2000). Limited Role of Direct Exchange Arthroplasty in the Treatment of Infected Total Hip Replacements. Clinical Orthopaedics and Related Research.

[27] Marculescu CE, Cantey JR (2008). Polymicrobial prosthetic joint infections: risk factors and outcome. Clin Orthop Relat Res.

[28] Deelstra JJ, Neut D, Jutte PC (2013). Successful treatment of *Candida albicans*-infected total hip prosthesis with staged procedure using an antifungal-loaded cement spacer. J Arthroplasty.

